# Drivers and Barriers for Plant-Based Cheese Alternatives Adoption: Insights from Diverse Consumer Clusters

**DOI:** 10.3390/foods14071162

**Published:** 2025-03-27

**Authors:** Marloes D. Schimmel, Jonas Yde Junge, Niki Alexi, Glenn Birksø Hjorth Andersen, Marianne Hammershøj, Mette Hadberg Løbner, Ulla Kidmose

**Affiliations:** Department of Food Science, Aarhus University, Agro Food Park 48, 8200 Aarhus N, Denmark; mds@food.au.dk (M.D.S.); jonas.junge@food.au.dk (J.Y.J.); niki.alexi@food.au.dk (N.A.); gha@food.au.dk (G.B.H.A.); marianne.hammershoj@food.au.dk (M.H.); mhl@food.au.dk (M.H.L.)

**Keywords:** survey, consumer, drivers and barriers, plant-based alternatives, cheese, dairy

## Abstract

The transition to plant-based diets is advocated as a consumption measure to mitigate the environmental impacts of animal-based food production. Understanding the drivers and barriers to consumption can guide the formulation of tailored strategies for advancing plant-based alternatives in markets. This study investigated the principal drivers and barriers influencing the adoption of plant-based cheese alternatives among Danish consumers (*n* = 550) through an online survey. Participants were clustered based on the sensory (flavor and texture) cues using Agglomerative Hierarchical Clustering, resulting in four consumer groups: (C1) consumers who prefer plant-based cheese alternatives to closely mimic both the flavor and texture of dairy cheese (*n* = 172); (C2) Consumers who prefer dairy-like flavor but are open to plant-based textures (*n* = 141); (C3) Consumers who prefer dairy-like texture but are open to novel flavor (*n* = 146); and (C4) Consumers who seek variety and novelty in both flavor and texture (*n* = 91). The results showed that consumer preferences for flavor and texture cues are important factors in shaping their motivations and barriers toward plant-based cheese. Specifically, product availability emerged as a significant barrier for those preferring dairy-like alternatives, while sensory perception and convenience were less influential for consumers who favor novelty. Notably, the consumer clusters did not show significant statistical differences in dietary pattern types, such as omnivores, flexitarians, vegetarians, or vegans. Understanding these dynamics is essential for developing effective strategies to promote plant-based cheese alternatives and cater to varying consumer needs.

## 1. Introduction

The transition towards plant-based diets offers a sustainable approach to global food consumption. In addition to animal welfare, this transition is primarily driven by increased consumer awareness of the environmental impacts associated with animal-based food production [1,2,3]. However, it presents significant challenges. These challenges include ensuring the nutritional adequacy of plant-based diets, establishing robust supply chains and production infrastructure for plant-based alternatives, and, most importantly, addressing consumer needs and acceptance as well as market dynamics [4]. Nutritional adequacy refers to the ability of plant-based alternatives to provide sufficient essential macro- and micronutrients, including high-quality proteins, vitamins such as B12 and D, essential minerals such as calcium and iron, and a well-balanced fatty acid profile. These nutrients are typically abundant in animal-based products but may require fortification or dietary diversification in plant-based alternatives to ensure adequate intake [5,6].

The increasing adoption of plant-based alternatives in Europe, including in Denmark [7,8,9,10], signals a shift towards more sustainable agricultural practices. This transition towards increased plant-based agricultural production could not only position European companies as significant players in the market for plant-based foods but also serve as a model for inspiring similar transitions globally [7]. By effectively addressing these challenges, the transition to plant-based diets can contribute to more sustainable food systems and mitigate the environmental impacts of traditional animal production.

The rising consumer interest in plant-based alternatives has created a target market that needs to cater to both vegetarian and vegan consumers but also aims to accommodate the demands of consumers who wish to reduce their animal-based food consumption [11]. While the plant-based cheese market is still in its early development in Denmark, the sales of plant-based cheese alternatives have increased by 150% in Germany, 165% in the United Kingdom, and 400% in the Netherlands from 2018 to 2020 [11,12].

The concept of novelty in food introduces a barrier in the transition towards plant-based alternatives. According to the European Commission, novel foods encompass newly developed, innovative products produced using new technologies and/or production processes, such as plant-based alternatives [13,14]. While, under appropriate conditions, such foods may represent a more sustainable diet, their novelty and unfamiliarity often pose a significant challenge to consumer acceptance.

Consumer preferences for plant-based alternatives vary significantly, particularly between those seeking novel, unique products and those desiring close mimics of animal-based counterparts. Flexitarians, who occasionally consume animal products but primarily follow a plant-based diet, often prefer plant-based alternatives that closely resemble traditional animal-based foods in flavor, texture, and appearance [15]. Conversely, some consumers, particularly those following strict plant-based diets, may prefer products with distinct sensory characteristics that do not attempt to replicate animal-based foods [16]. These different preferences present a challenge for product developers in the plant-based sector as they must cater to both novelty-seeking consumers and those preferring familiar alternatives.

Transitioning to plant-based alternatives, in the context of cheese alternatives, presents unique challenges to consumers and the food industry. Unlike traditional cheese-making processes involving fermentation and maturation, plant-based alternatives are crafted from a blend of oils, fibers, plant-based proteins, and starches. This leads to products with different sensory profiles that many consumers find sensorily unacceptable because of a lack of cheesy flavor and bitterness, creamy textures, and natural aromas [11,17,18,19]. Consequently, while consumer interest in plant-based options continues to rise, their acceptance remains limited. It has been suggested that promoting plant-based food consumption can be achieved by designing new products that meet consumers’ sensory preferences and align more closely with their beliefs and lifestyle choices [20]. Both subjective and objective knowledge levels may play a role in driving food choices, leading consumers to select foods that align with their beliefs rather than exploring alternative options [21].

Studies have indicated that younger generations, often described as Gen Z (18–29 years of age) and Millennials (30–41 years of age), tend to be more willing to adopt a diet containing plant-based alternatives than animal-based products [22]. Our study aims to understand the underlying factors that drive or hinder the adoption of plant-based cheese in Danish Gen Z and Millennial consumers’ diets, thus facilitating targeted promotional efforts for plant-based food alternatives. While previous research has explored consumer attitudes toward plant-based alternatives, studies specifically on plant-based cheese remain limited. This study focuses on individuals who already incorporate such alternatives into their diets regularly or intend to increase their intake of plant-based alternatives in the future, providing a more comprehensive understanding of the overall dietary habits, needs, and preferences of this target group. The primary aim is to identify drivers and barriers that should be addressed to enhance plant-based cheese alternative intake. Moreover, we aim to investigate potential differences in these factors among consumer clusters with different product preferences, providing valuable insights into tailored strategies for product development and marketing. This research holds significant value for the Danish food industry and other markets with similar characteristics as it seeks to inform the reformulation of plant-based cheese alternatives, addressing consumer needs and preferences effectively.

## 2. Materials and Methods

### 2.1. Study Participants

This study included respondents (*n* = 550) between the ages of 18 and 41 (Gen Z and Millennials), proficient in Danish, and either regular purchasers (at least monthly) of plant-based cheese alternatives (*n* = 469; 85.3%) or intended to increase their consumption of plant-based alternatives to dairy and/or cheese in the future (any intended increase amount was included; *n* = 81; 14.7%).

Our recruitment process ensured a balanced sample in terms of gender and age, with quotas set for both the 18–29 and 30–41 age categories. Additionally, regional recruitment quotas were implemented. To ensure the integrity of our dataset, we conducted a thorough data check. Respondents who failed attention checks within the online questionnaire or did not complete the questionnaire in its entirety were excluded from subsequent analysis. The screening criteria were based on consumption (or intended consumption) of plant-based cheese alternatives rather than general plant-based consumption. An external agency was engaged to conduct the programming and deployment of the survey to achieve the established quotas. The survey was deployed in 2022 and took participants approximately 10–15 min to complete.

Respondent characteristics are displayed in Table 1. Most of the respondents were female (70%). The respondents had an average age of 29 years and predominantly resided in bigger cities (61%). Regarding culinary responsibilities, 50% reported being jointly responsible, followed by 41% solely responsible for household cooking. Dietary preferences varied, with the majority adhering to an omnivorous diet (41%) or a flexitarian diet (35%).

### 2.2. Study Procedure

The questionnaire used in this study was an adapted version of the questionnaire developed for the European Institute of Innovation and Technology (EIT)-funded multi-country project “The V-PLACE—Enabling consumer choice in vegan or vegetarian food products” [23]. The questionnaire was divided into different sections. The respondents were asked to report their intake frequency of different products, including hard cheese (e.g., parmesan), semi-hard cheese (e.g., Danbo or Samsø), cream cheese, pizza-topping cheese, and plant-based alternatives to these items. This was performed using a 7-point Likert scale ranging from “Never” to “Daily”.

In the next section, the questionnaire inquired about product preferences for semi-hard plant-based cheese alternatives in terms of flavor, texture, and nutritional content. Preferences for flavor and texture were evaluated through single-choice questions, while preferences for nutritional contents were assessed using the Check-All-That-Apply (CATA) method. The answer options for flavor preference included “Should have a flavor mainly like their plant-derived ingredient (e.g., almond flavor for almond-based cheese)”, “Should have flavor like their corresponding dairy-based product (e.g., dairy-based semi-hard cheese)”, “Should have a hint of the plant-based ingredient but not be overwhelming (e.g., mild almond flavor for almond-based cheese)”, “Should be a new/alternative flavor experience”, “I prefer different flavor variations”, and “Flavor is not important to me”. For texture preference, the answer options included “The texture should be exactly the same as the conventional dairy-based product (e.g., exactly the same softness for the plant-based alternative to semi-hard cheese as in dairy-based semi-hard cheese)”, “The texture should be similar to the conventional dairy-based product but does not need to be exactly the same (e.g., similar softness for the plant-based alternative to semi-hard cheese as in dairy-based semi-hard cheese)”, “It should be a new/alternative texture”, “I prefer different textures”, and “Texture is not important to me”. For nutritional preference, the participants could select multiple nutritional attributes they valued, namely “High protein content”, “Enrichment with minerals (calcium)”, Enrichment of vitamins (B12, vitamin D)”, “Low calorie”, “Low-fat”, “Low-salt content”, and “A balanced micro-macro nutrient profile”, or they could indicate that none of these factors were important to them, or specify other preferences.

The third section of the questionnaire aimed to understand the drivers and barriers to selecting plant-based alternatives to cheese. The respondents evaluated 20 drivers (“Benefits to my health”, “Is beneficial for my well-being”, “Brings variety to my diet”, “Less environmental impact”, “Social awareness including human rights and justice”, “Animal welfare”, “Without additives”, “Produced naturally”, “Flavor”, “Consistency”, “Texture”, “Mouthfeel”, “Availability in the market”, “Dietary habits of social circle”, “Religion”, “Trends’’, “Lifestyle”, and “Popularity”). This was performed using a 5-point Likert scale ranging from “Not important at all” to “Very important”.

In the next section of the questionnaire, the questions that aimed to understand potential barriers to selecting plant-based alternatives to cheese were categorized differently; respondents evaluated 20 barriers (“Product variability”, “Availability in market/restaurants/shops”, “Flavor”, “Mouthfeel”, “Nutritional deficiencies”, “Intolerance/Allergies”, “High price”, “Excessive additives/preservatives”, “High in sugar”, “High salt content”, “Excessive processing”, “Extended preparation time”, “Perceived healthiness”, “Sustainability”, “Trustworthiness of labels”, “Lack of knowledge about nutritional profiles”, “Non-local production”, “Non-organic products”, “Ability to share with family/friends/community”, and “Feelings of being understood and acceptance of the choice”). The respondents indicated their level of agreement or disagreement with each statement using a 5-point Likert scale ranging from “Strongly Disagree” to “Strongly Agree”.

The last section of the questionnaire consisted of questions about consumers’ socioeconomic demographics and lifestyle context, i.e., age, gender, education level, diet (whether consumers identified themselves as an omnivore, flexitarian, pescatarian, vegetarian, or vegan), income, type of area living in, and who is responsible for cooking in the household.

### 2.3. Data Analysis

All statistical analyses were conducted using R (version 4.3.2) [24] and RStudio (version 2023.12.1) [25]. Demographic information was summarized using the R package tableone (https://CRAN.R-project.org/package=tableone, accessed on 13 February 2025) [26]. Unless otherwise stated, visualizations are conducted using the ggplot2 [27] and tidyverse [28] packages. All statistical tests are conducted using α = 0.05.

The cluster analysis was conducted using Agglomerative Hierarchical Clustering with Gower’s distances and Wards method using the R package cluster (https://cran.r-project.org/web/packages/cluster/index.html, accessed on 13 February 2025) [29]. Cower’s distances were calculated based on consumers’ product preferences for plant-based alternatives to semi-hard cheeses among factors: (i) flavor and (ii) texture, which were treated as nominal data, and (iii) nutrition treated as sets of binary data. As the question type varied for the three factors, they were weighted, so each factor (flavor, texture, and nutritional content) had the same weight in the clustering. Using the R package NbClust [30], the optimal number of clusters using the majority rule was 5. However, to have sufficient (>50) participants in each cluster, 4 clusters were chosen for further analysis. The *p*-values for differences between clusters were identified from an ANOVA test using the R package tableone. Significant differences between clusters in product preferences were found using Fisher’s Exact Test using rstatix [31].

The items identifying participants’ drivers and barriers were grouped into broader concepts with Exploratory Factor Analysis (EFA) from lavaan [32] with a cut-off of 0.35. The drivers and barriers were analyzed separately. The differences between EFA concepts were subsequently compared between clusters using Tukey’s post hoc test for pairwise comparisons with the emmeans package [33].

### 2.4. Ethical Considerations

In Denmark, ethical approval is not required for non-health related survey studies according to the National Committee on Health Research Ethics in Denmark (Section 14 (2) in the Committee Act) (Bekendtgørelse af lov om videnskabsetisk behandling af sundhedsvidenskabelige forskningsprojekter [34]). Informed ethical consent was obtained from all respondents involved in the study.

## 3. Results

### 3.1. Cluster Profiles

**Figure 1:**
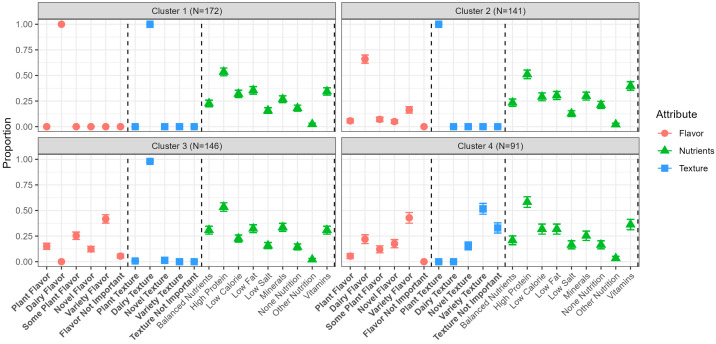
Selection proportions of terms for preferences related to flavor, texture, and nutritional cues of plant-based cheese alternatives across the four respondent clusters. Flavor factors are marked red circles, texture factors are marked blue squared, and nutritional factors are marked with green triangles. Statistically significant factors by Fisher’s Exact Test (*p* < 0.05) across the clusters are indicated by bold font.

Using Agglomerative Hierarchical Clustering on the product preferences for plant-based cheese alternatives, four consumer clusters were identified. Their differences in product preferences can be seen in Figure 1. The clusters primarily differed in the respondents’ preferences for flavor and texture attributes related to dairy cheese and plant-based alternatives. In C1, a larger proportion of consumers preferred plant-based cheese alternatives to have a flavor and texture similar to cheese compared with the other clusters. Consumers in C2 showed a stronger preference for plant-based cheese alternatives that resemble cheese flavor while retaining the texture characteristic of plant-based cheese alternatives compared with other clusters. Consumers in C3 preferred a texture similar to cheese, but the majority of this cluster favored a flavor different from cheese in contrast to C1 and C2. A higher proportion of consumers in C4 preferred a greater variety in flavor and texture compared with other clusters. The different clusters C1–C4 are illustrated in Figure 1.

Regarding the nutritional cues, the results showed that high protein content is prioritized across all clusters (C1, C2, C3, and C4) for plant-based cheese alternatives, alongside other nutritional benefits (Figure 1). A shared trait among all clusters was a high preference for low-calorie, low-fat options and added vitamin content (vitamin B12/D). Conversely, there was a diminished preference for low-salt options and disinterest in additional nutritional fortification across the clusters.

**Figure 2:**
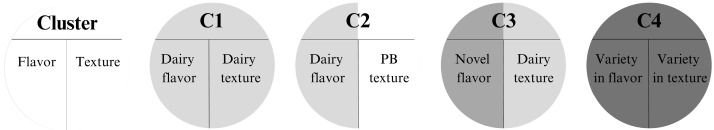
Illustration of the clustering of respondents based on their preferences for flavor and texture of plant-based cheese alternatives. The different colors indicate different preference characteristics among the clusters, namely: white: plant-based; light grey: dairy; medium grey: novelty; dark grey: variety.

As illustrated in Figure 2, we observed that consumers in C1 preferred dairy flavor and texture, whereas C4 exhibited a contrasting inclination towards variety-seeking behavior. Clusters C2 and C3 fall in between the two others. Where consumers in C2 prefer plant-based cheese alternatives to resemble dairy in flavor but the plant-based ingredient in texture, consumers in C3 prefer plant-based cheese alternatives to have a texture similar to their dairy counterparts but a novel flavor.

### 3.2. Cluster Socio-Demographics and Consumption Patterns of Dairy and Non-Dairy Alternatives

The different respondent characteristics among the four identified clusters are displayed in Table 2. No significant differences were found in any of the characteristics shown in Table 2.

**Figure 3:**
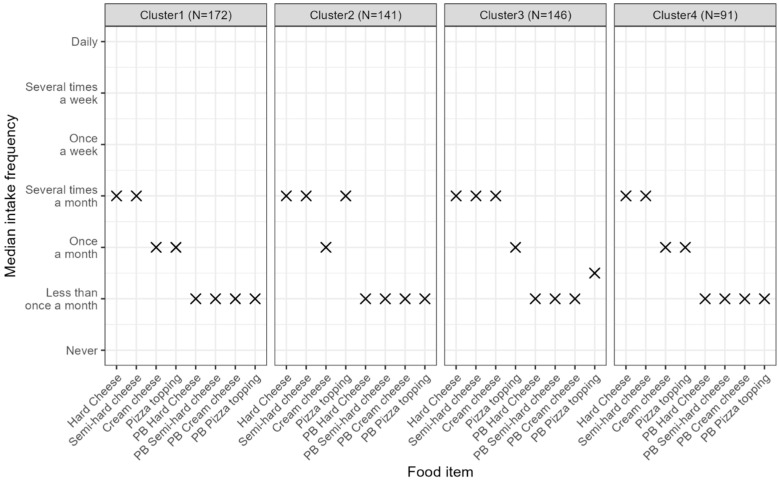
Median (indicated with x) intake frequencies of dairy and plant-based alternatives to cheeses across the four clusters of respondents. C1: Dairy flavor; dairy texture (*n* = 172); C2: Dairy flavor; PB texture (*n* = 146); C3: Dairy texture; novel flavor (*n* = 141); and C4: Variety in flavor; variety in texture (*n* = 91).

As shown in Figure 3, the frequency of cheese consumption was notably similar across all clusters, with hard and semi-hard cheeses being the most consumed types, with a median intake of several times per month in all clusters. Consumption of plant-based cheese alternatives was consistently lower compared with cheese across all clusters, with median intake reported to “Less than once a month” for most plant-based cheese alternatives categories in all clusters.

### 3.3. Drivers for Plant-Based Cheese Alternatives Across Different Clusters

The 20 drivers evaluated by the respondents were grouped using Exploratory Factor Analysis. The optimal number of factors was determined to be five. These factors were related to different aspects drivers, namely Health (“Benefits to my health”, “Is beneficial for my well-being”, and “Brings variety to my diet”), Ethics (“Less environmental impact”, “Social awareness including human rights and justice”, and “Animal welfare”), Naturalness (“Without additives” and “Produced naturally”), Sensory and Convenience (“Flavor”, “Consistency”, “Texture”, “Mouthfeel”, and “Availability in the market”), and (social) Influence aspects (“Dietary habits of social circle”, “Religion”, “Trends’’, “Lifestyle”, and “Popularity”). Table 3 shows the factors Health concerns, Ethical concerns, Naturalness, and Sensory and Convenience were identified as drivers for consumers to adopt plant-based cheese alternatives into their diet, as their mean ratings are above the neutral point on the scale (‘Neither important nor not important’). On the contrary, Social influence, as a whole, was the least important factor. This study showed that the effects of the Sensory and Convenience factors, as well as Social influence, differed among clusters. We observed that consumers who wished for different textures than dairy (C2 and C4) place less importance on Sensory and Convenience, whereas those who preferred a cheese texture (C1: Dairy flavor; dairy texture) exhibited a higher influence from sensory perception. Additionally, we observed that consumers with a preference for a higher degree of variety (C4: variety in flavor; variety in texture) are more driven by Social influences, e.g., dietary habits of peers, religion, trends, lifestyle and popularity compared with consumers who do not prefer food novelty (C1: dairy flavor; dairy texture).

### 3.4. Barriers to Consuming Plant-Based Cheese Alternatives Among Different Clusters

Similar to drivers, the 20 barriers evaluated by the respondents were grouped using Exploratory Factor Analysis. Here, the optimal number of factors was determined to be four, and the factors were barriers related to Availability (“Product variability”, and “Availability in market/restaurants/shops”), Sensory Experience (“Flavor” and “Mouthfeel”), Health and Sustainability (“Nutritional deficiencies”, “Intolerance/Allergies”, “High price”, “Excessive additives/preservatives”, “High in sugar”, “High salt content”, “Excessive processing”, “Extended preparation time”, “Perceived healthiness”, “Sustainability”, “Trustworthiness of labels”, “Lack of knowledge about nutritional profiles”, “Non-local production”, and “Non-organic products”), and Socials Influence aspects (“Ability to share with family/friends/community” and “Feelings of being understood and acceptance of the choice”). Table 4 shows the perceived barriers associated with plant-based cheese alternative consumption across clusters. The factor Availability (including product variability) in supermarkets/restaurants was identified as a barrier among all clusters; however, the effect was more pronounced for consumers who had a preference for products with a dairy-like texture and exhibited lower inclinations toward food novelty. We observed that Sensory and Convenience aspects were not a barrier for C4 (variety in flavor; variety in texture), who sought variety and novelty, while we identified it as a barrier among C1 (dairy flavor; dairy texture), which was a cluster of consumers preferring traditional characteristics of plant-based alternatives to cheese. Additionally, it is evident that Health and Sustainability considerations and the impact of Social influence on consuming plant-based cheese alternatives were not regarded as barriers across the different clusters (values < 3.0).

## 4. Discussion

Our study evaluated the factors influencing the adoption of plant-based cheese alternatives among Danish Gen Z and Millennial consumers, aiming to identify drivers and barriers that must be addressed to enhance the consumption of such products among consumers in those age categories. We investigated whether these factors vary among individuals with different product preferences to inform targeted strategies for developing and marketing plant-based food alternatives. Through consumer clustering, we showed that consumers with varying preferences for plant-based cheese alternatives also differed in their barriers and drivers for consuming these products. Specific preferences for both flavor and texture were a major part of this.

### 4.1. Drivers and Barriers Across All Clusters

Among Danish consumers who regularly consume plant-based cheese alternatives, we found that health and sustainability considerations, coupled with a wish for high-protein content, were drivers across all clusters. In Europe, more and more plant-based products that are designed to mimic their animal-sourced counterparts are launched on the market. Increased demand for plant-based products highlights concerns regarding the nutritional profile of existing plant-based cheese alternatives, particularly the gap between consumer preferences and actual protein content [35,36]. Despite half of the consumers in our study preferred high protein options, most products fail to meet this expectation, emphasizing the need to address this concern.

While consumers may acknowledge the potential health benefits of plant-based diets, they may not automatically view plant-based alternatives as healthier or more environmentally friendly than animal-based products [37]. To explore this further, we turned to the concept of Naturalness. In our study, it was observed that Naturalness drove all consumer clusters strongly, thus prioritizing products free from artificial additives or with clean labels. This aligns with previous research, which found that consumers seek convenient, tasty, plant-based products with a simple ingredient list [4]. In a study with Danish consumers, it was indicated that individuals who perceive plant-based milk alternatives as highly processed or artificial were less inclined to accept them compared with individuals who perceive them as natural and minimally processed [38]. This observation could likewise be extended to plant-based cheese alternatives. An explanation for this might be that the consumers associate such products with lower quality and health benefits compared with the less processed options. This finding is consistent with other studies, which, in general, have found unprocessed foods to be perceived as healthier when compared with their processed counterparts [39,40]. While consumers prioritize naturalness in their food choices, efforts to enhance the nutritional profile of plant-based cheese alternatives through fortification present a challenge, as fortification may compromise naturalness and ultimately impact consumer acceptance [41,42]. Hence, efforts to enhance the nutritional profile of plant-based cheese alternatives must carefully balance health considerations with consumer desires for naturalness to effectively promote acceptance in the market.

### 4.2. Different Cluster Preferences and Their Cheese Consumption Pattern

Among the different clusters, two exhibited contrasting preferences: Consumers in C1 preferred cheese-like flavor and textures, while consumers in C4 expressed a preference for a greater variety and, thereafter, novelty in flavor and texture profiles. However, this did not translate into consumption patterns, as C1 and C4 did not differ in intake frequency of different types of plant-based alternatives to cheese. This is in contrast to previous research by Hoek, et al. [43], who suggested that repeated experiences influence consumer acceptance of plant-based meat alternatives. In a sample of 89 Dutch participants, they found that the more often participants consumed plant-based meat alternatives, the less similar to meat they wanted the alternatives to be in the sensory traits.

### 4.3. Major Drivers and Barriers Between the Different Clusters

Mimicking both cheese flavor and texture in plant-based cheese alternatives has proven challenging [11,17,18,19,44,45,46,47,48]. Our study indicated that preferences for both the flavor and texture of plant-based cheese alternatives vary greatly across consumers, driving the clustering.

Within the different clusters, we observed that the degree to which the flavor and texture experience is a barrier differed. The sensory experience was not a barrier for consumers in C4 (Variety in flavor; variety in texture), while we identified it as a barrier among consumers in C1 (Dairy flavor; dairy texture). This might relate to our finding that consumers in C1 find Availability as a consumption barrier and Sensory perception and Convenience as drivers, both to a larger degree than consumers in C4. For consumers in C1, availability might refer to the lower availability of products with sensory properties mimicking the conventional products they prefer. Similarly, when consumers wish for variety and novelty in flavor and texture, as is the case for consumers in C4, they tend to find Availability less of a barrier and Sensory perception and Convenience as less of drivers. This might simply occur because most plant-based cheese alternatives are as they prefer, and thus, both are more available and have the sensory properties they prefer. Interestingly, our findings showed no significant differences in dietary habits (omnivores, flexitarians, vegetarians) between C1 and C4. Notably, previous studies have suggested omnivores and flexitarians might prefer plant-based alternatives with sensory properties similar to animal-based counterparts. Studies by Michel, et al. [49] and Hoek, et al. [50] found that omnivores and flexitarians showed a stronger preference for plant-based meat alternatives that closely mimicked the flavor and texture of meat.

While strategies such as increasing availability and targeted marketing campaigns may offer some benefits [51], our findings suggest that addressing the diverse preferences of consumer clusters requires a more nuanced approach, potentially incorporating innovative product development and tailored marketing strategies.

Additionally, we observed a slight but not significant difference in consumers with a preference for food variety and novelty (C4) that are more driven by Social influences, e.g., dietary habits of peers, religion, trends, lifestyle, and popularity compared with consumers who prefer dairy flavor and texture (C1). Consumers in C4, driven by their inclination toward variety and novelty, may be more influenced by social factors such as dietary habits among their peers, religious beliefs, lifestyle trends, and product popularity. This suggests that their food preferences and acceptance are shaped not only by sensory experiences but also by external influences, including social norms. Other studies indicated that social norms play a crucial role in shaping consumer acceptance of alternative proteins, with multiple studies consistently highlighting the influence of the social environment on the acceptance of these products [52,53,54]. For example, negative opinions from family and friends have been identified as significant barriers to trying alternative proteins such as pulses [55]. In contrast, consumers in C1, who lean toward familiar dairy-like flavors and textures, seem to prioritize familiarity and expectations in their food choices. They may exhibit less openness to novel foods because of their inherent preference for what is known and familiar. Notably, this understanding of the complex dynamics shape consumer acceptance of plant-based foods in general [13] and cheese specifically [56].

### 4.4. Strengths, Limitations, and Future Research

The strengths of this study are that we focused solely on individuals who consumed or intended to increase their consumption of plant-based cheese alternatives. We included questions about other plant-based alternatives, which provided a more comprehensive understanding of consumers’ overall dietary habits and preferences.

While our study offers insights into consumer drivers and barriers among plant-based cheese alternatives, some limitations are acknowledged. First, there was a gender imbalance among respondents, with 70% identifying as female. This overrepresentation of female respondents could have biased the findings by amplifying drivers and reducing barriers typically associated with plant-based product adoption, as previous research indicates that women are generally more open to adopting plant-based products compared with men [57].

Second, consumers were divided into different clusters with different characteristics in flavor, texture, and nutritional preferences. However, the clusters did not differ in any of the socioeconomic and demographic characteristics (e.g., dietary preferences) and had overlapping characteristics of flavor, texture, and nutritional preferences, therefore limiting the generalizability of our findings and the extent to which they can be applied to broader consumer populations. This is especially true as the study only included consumers already consuming or intent to increase plant-based alternative consumption. Another limitation of the methodological approach was that we used Exploratory Factor Analysis to group the survey questions in which the term naturalness was grouped differently among driver- and barrier-related questions. When identifying drivers for consumption of plant-based cheese alternatives, the term “Naturalness” referred in the questionnaire to ‘produced naturally’, and ‘without additives’ and was observed as a factor itself, whereas when identifying barriers for plant-based cheese consumption, the term naturalness was categorized under health and environmental concerns. This statistical approach has led to inconsistency and variability in grouping the questions. Acknowledging this limitation highlights the need for a more consistent future survey design to ensure the validity of study findings, providing large questionnaires among consumers.

## 5. Conclusions

Our study aimed to investigate the primary drivers and barriers influencing the adoption of plant-based cheese alternatives among Danish consumers. We identified four different consumer clusters based on their preferences for plant-based cheese alternatives: (C1) Consumers who prefer plant-based cheese alternatives to closely mimic both the flavor and texture of dairy cheese; (C2) Consumers who prefer dairy-like flavor but are open to plant-based textures; (C3) Consumers who prefer dairy-like texture but are open to novel flavors; (C4) Consumers who seek variety and novelty in both flavor and texture. These clusters showed different barriers and drivers for consuming plant-based cheese alternatives. The availability was a significant barrier for consumers in C1, who preferred dairy-like alternatives, while it was less of a concern for those in C4, who preferred variety and novelty. Sensory perception and convenience were stronger drivers for C1, whereas C4 was more influenced by social factors. Although we found no significant differences in dietary habits (omnivores, flexitarians, and vegetarians) between the clusters, understanding the consumers’ drivers and barriers is crucial for reformulating plant-based cheese alternatives to meet the diverse preferences of consumers. In summary, addressing both the sensory qualities of flavor and texture, as well as the need for a high-protein content, could help improve the acceptance of these products across different consumer segments. Furthermore, focusing on clean-label formulation and minimizing artificial additives can enhance consumer trust in plant-based cheese alternatives. Future research on the willingness of consumers with different dietary preferences to embrace plant-based cheese alternatives would highlight difficulties in shifting to more plant-based consumption. This could lead to a better understanding of the main reasons for adopting plant-based cheese into the diet. This information could then be leveraged to promote a broader shift towards plant-based alternatives, particularly within food product categories that are deemed unsustainable when consumed excessively.

## Figures and Tables

**Table 1:** Socioeconomic-demographic characteristics of total sample (*n* = 550). The percentages have been rounded up to the nearest integer for interim calculations.

Variable	Total (*n* = 550)	Percent (%)
*Gender*		
Female	383	70
Male	162	30
Age (mean)	29	
*Income (DKK)*		
<100,000	54	12
100,001 to 400,000	41	9
500,001 to 900,000	210	45
>900,000	164	35
*Cooking*		
Me	227	41
Someone else	50	9
Together	273	50
*Diet type*		
Omnivore	240	44
Flexitarian	191	35
Pescatarian	44	8
Vegetarian	30	6
Vegan	45	8
*Education level*		
Elementary school	42	8
Vocational training	49	9
High school	151	28
Courses, no exam/degree	1	0
Master’s degree	122	22
PhD degree	3	1
Short higher degree	178	33
*Type of habitant area*		
Capital/big city	334	61
City (>20,000 habitants)	111	20
Village (<20,000 habitants)	103	19

**Table 2:** Socio-demographic characteristics across clusters are shown in numbers of respondents and percentages in brackets. C1: Dairy flavor; dairy texture (*n* = 172); C2: Dairy flavor; PB texture (*n* = 146); C3: Dairy texture; novel flavor (*n* = 141); and C4: Variety in flavor; variety in texture (*n* = 91). The clusters did not show statistically significant differences in the variables.

Variable	C1 Dairy Flavor; Dairy Texture	C2 Dairy Flavor; PB Texture	C3 Novel Flavor; Dairy Texture	C4 Variety in Flavor; Variety in Texture
*Gender*				
Female	121 (71%)	105 (73%)	105 (75%)	52 (58%)
Male	50 (29%)	39 (27%)	36 (25%)	37 (42%)
Age (mean)	28	29	28	30
*Income (DKK)*				
<100,000	19 (13%)	16 (13%)	10 (8%)	9 (11%)
100,001 to 400,000	64 (44%)	45 (37%)	59 (49%)	42 (52%)
500,001 to 900,000	53 (36%)	48 (40%)	40 (33%)	23 (28%)
>900,000	11 (8%)	12 (10%)	11 (9%)	7 (9%)
*Cooking*				
Me	69 (40%)	59 (40%)	63 (45%)	36 (40%)
Someone else	10 (6%)	14 (10%)	15 (11%)	11 (12%)
Together	93 (54%)	73 (50%)	63 (45%)	44 (48%)
*Diet type*				
Omnivore	72 (42%)	58 (40%)	70 (50%)	40 (44%)
Flexitarian	53 (31%)	63 (43%)	44 (31%)	31 (34%)
Pescatarian	20 (12%)	7 (5%)	10 (7%)	7 (8%)
Vegetarian	16 (9%)	9 (6%)	8 (6%)	12 (13%)
Vegan	11 (6%)	9 (6%)	9 (6%)	1 (1%)
*Education level*				
Elementary school	19 (11%)	14 (10%)	3 (2%)	6 (7%)
Vocational training	12 (7%)	13 (9%)	15 (11%)	9 (10%)
High school	29 (29%)	35 (24%)	38 (27%)	29 (32%)
Courses, no exam/degree	0	0	1 (1%)	0
Master’s degree	41 (24%)	33 (23%)	30 (21%)	18 (20%)
PhD degree	0	2 (1%)	0	1 (1%)
Short higher degree	51 (30%)	47 (33%)	53 (38%)	27 (30%)
*Type of habitant area*				
Capital (>1,000,000)	114 (66%)	87 (60%)	77 (55%)	56 (62%)
City (>20,000)	29 (17%)	29 (20%)	36 (26%)	17 (19%)
Village (<20,000)	29 (17%)	29 (20%)	27 (19%)	18 (20%)

**Table 3:** Means and confidence intervals of rated importance of consumer drivers for selecting plant-based alternatives to cheese across distinct respondent clusters. Distinct clusters include C1: Dairy flavor; dairy texture (*n* = 172); C2: Dairy flavor; PB texture (*n* = 146); C3: Dairy texture; novel flavor (*n* = 141); and C4: PB flavor; novel texture (*n* = 91). Values range from Not Important (1) to Neutral (3) to Very Important (5). Bold, shaded values indicate significant differences (light grey: a; medium grey: ab; dark grey: b). CI: 95% Confidence Interval.

	C1 Dairy Flavor; Dairy Texture		C2 Dairy Flavor; PB Texture		C3 Novel Flavor; Dairy Texture		C4 Variety in Flavor; Variety in Texture	
	Mean	CI	Mean	CI	Mean	CI	Mean	CI
Health	3.74	3.63–3.85	3.84	3.73–3.96	3.81	3.70–3.93	3.80	3.66–3.95
Ethical	4.17	4.05–4.29	4.14	4.01–4.27	3.98	3.85–4.12	4.16	3.99–4.33
Naturalness	3.87	3.74–3.99	3.88	3.74–4.02	3.89	3.74–4.03	3.91	3.73–4.08
**Sensory and Convenience**	**4.11 ^ab^**	4.02–4.19	**4.04 ^a^**	3.95–4.14	**4.25 ^b^**	4.15–4.34	**4.02 ^a^**	3.90–4.13
**Social influence**	**2.04 ^a^**	1.91–2.17	**2.15 ^ab^**	2.01–2.29	**2.16 ^ab^**	2.02–2.30	**2.41 ^b^**	2.23–2.58

**Table 4:** Means and confidence intervals of rated agreement of consumers barriers for selecting plant-based alternatives to cheese across the distinct respondent clusters. Distinct clusters include C1: Dairy flavor; dairy texture (*n* = 172); C2: Dairy flavor; PB texture (*n* = 146); C3: Dairy texture; novel flavor (*n* = 141); and C4: Variety in flavor; variety in texture (*n* = 91). Values range from Strongly Disagree (1) to Neither agree or disagree (3) to Strongly Agree (5). Bold, shaded values indicate significant differences (light grey: a; medium grey: ab; dark grey: b). CI: 95% Confidence Interval.

	C1 Dairy Flavor; Dairy Texture		C2 Dairy Flavor; PB Texture		C3 Novel Flavor; Dairy Texture		C4 Variety in Flavor; Variety in Texture	
	Mean	CI	Mean	CI	Mean	CI	Mean	CI
**Availability**	**3.81 ^b^**	3.67–3.95	**3.73 ^ab^**	3.58–3.88	**3.78 ^b^**	3.63–3.94	**3.42 ^a^**	3.23–3.62
**Sensory experience**	**3.18 ^b^**	3.03–3.33	**2.83 ^a^**	2.66–2.99	**3.39 ^b^**	3.22–3.55	**2.71 ^a^**	2.51–2.92
Health and Sustainability	2.62	2.51–2.72	2.68	2.57–2.80	2.79	2.67–2.90	2.82	2.68–2.97
Social influence	2.20	2.03–2.36	2.29	2.11–247	2.42	2.23–2.60	2.48	2.25–2.71

## Data Availability

The data presented in this study are available on request from the corresponding author due to privacy.
